# Supplementary material for: SynthStrip: skull-stripping for any brain image
Source: Neuroimage. Author manuscript; Available in PMC 2022 Oct 15. (PMC9465771; doi:10.1016/j.neuroimage.2022.119474)
Supplement: 1 [file NIHMS1833287-supplement-1.zip › mmc1/supp_figure_1.pdf]

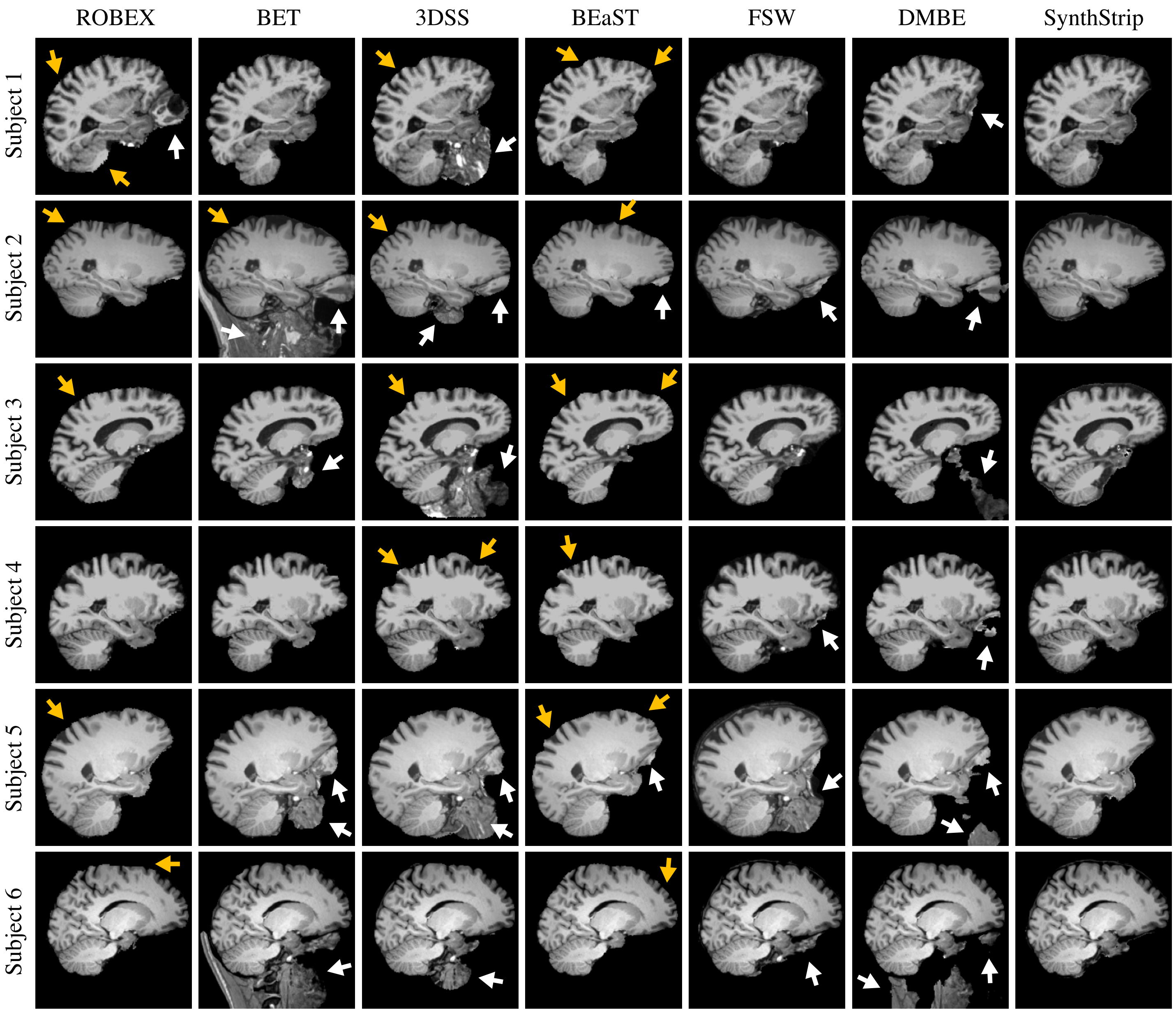

Figure S1. Comparison of representative skull-stripping errors for each method across six individual test scans. White arrows indicate over-labeling of the brain mask, while orange arrows indicate removal of brain matter.
