# Supplementary material for: SynthStrip: skull-stripping for any brain image
Source: Neuroimage. Author manuscript; Available in PMC 2022 Oct 15. (PMC9465771; doi:10.1016/j.neuroimage.2022.119474)
Supplement: 1 [file NIHMS1833287-supplement-1.zip › mmc1/supp_figure_2.pdf]

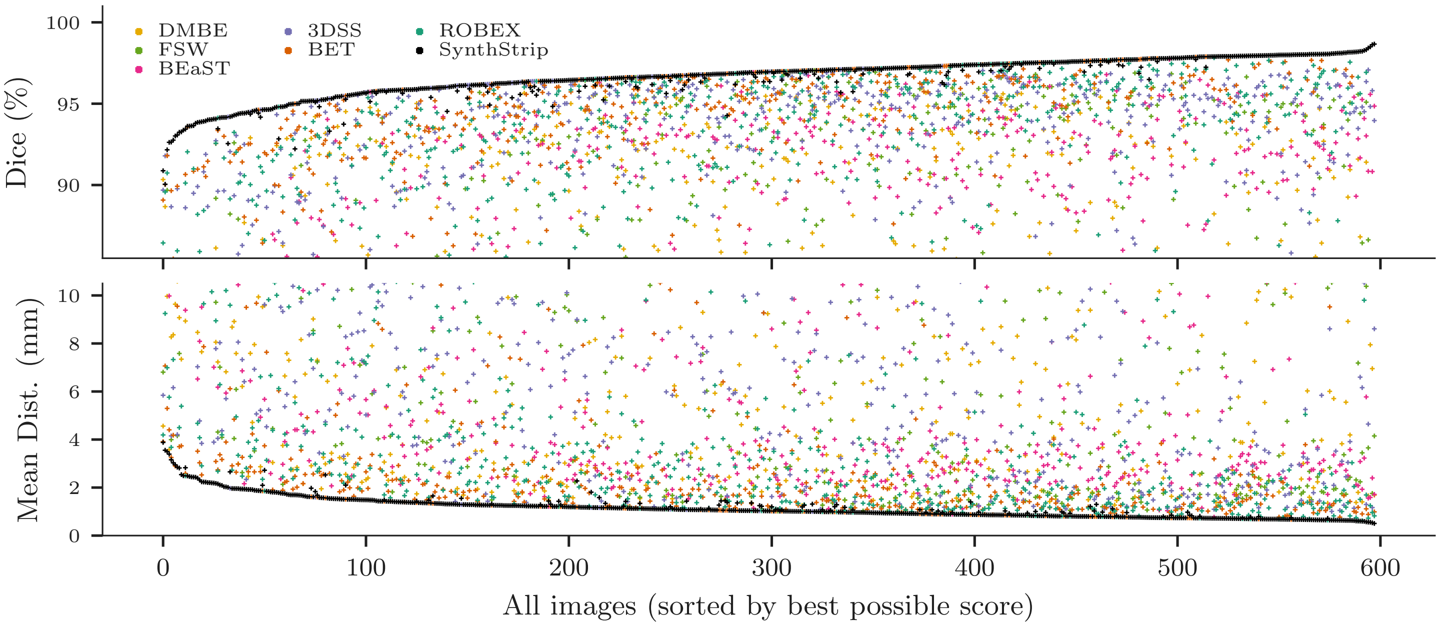

Figure S2. SynthStrip accuracy compared to baseline methods, across all images in the test set. Images are sorted by the score of the top performing skull-stripping method. Each dot represents a single brain mask derived with a particular tool, and each column of dots represents the scores obtained for a single image across tools.
