# Supplementary material for: SynthStrip: skull-stripping for any brain image
Source: Neuroimage. Author manuscript; Available in PMC 2022 Oct 15. (PMC9465771; doi:10.1016/j.neuroimage.2022.119474)
Supplement: 1 [file NIHMS1833287-supplement-1.zip › mmc1/supp_table_1.pdf]

Max Surface Distance (mm)

	SynthStrip	ROBEX	BET	3DSS	BEaST	FSW	DMBE
IXI T1w	13.5 ± 7.0	17.4 ± 6.8<br>3.9×10^{-11}	17.2 ± 11.8<br>2.0×10^{-2}	29.3 ± 13.2<br>1.2×10^{-9}	20.6 ± 6.1<br>5.7×10^{-22}	20.7 ± 17.1<br>8.1×10^{-4}	39.5 ± 27.1<br>1.8×10^{-8}
FSM T1w	12.3 ± 2.7	16.0 ± 2.2<br>6.1×10^{-9}	85.4 ± 22.5<br>1.9×10^{-20}	31.0 ± 14.4<br>2.7×10^{-9}	19.3 ± 2.3<br>2.9×10^{-16}	22.4 ± 15.6<br>3.4×10^{-4}	70.7 ± 26.3<br>9.1×10^{-16}
ASL T1w	12.2 ± 2.7	15.0 ± 3.6<br>2.5×10^{-4}	16.1 ± 4.4<br>1.8×10^{-5}	32.6 ± 14.7<br>7.0×10^{-11}	20.9 ± 5.4<br>1.3×10^{-12}	11.7 ± 1.4<br>2.2×10^{-1}	52.6 ± 20.9<br>5.4×10^{-16}
QIN T1w	16.9 ± 5.3	21.9 ± 6.0<br>3.0×10^{-5}	22.7 ± 7.2<br>5.6×10^{-7}	39.3 ± 12.4<br>1.5×10^{-17}	29.0 ± 19.1<br>2.3×10^{-5}	42.1 ± 20.1<br>1.8×10^{-12}	32.8 ± 10.0<br>1.3×10^{-15}
IXI T2w	13.1 ± 4.0	16.9 ± 4.8<br>1.2×10^{-6}	22.6 ± 7.7<br>6.9×10^{-11}	31.5 ± 13.6<br>1.8×10^{-12}	66.2 ± 21.5<br>5.9×10^{-23}	-<br>-	122.4 ± 17.7<br>3.6×10^{-39}
FSM T2w	11.2 ± 2.6	13.7 ± 4.8<br>9.9×10^{-3}	21.4 ± 8.9<br>1.8×10^{-7}	22.9 ± 9.9<br>4.5×10^{-8}	59.2 ± 29.2<br>2.0×10^{-11}	-<br>-	136.9 ± 21.8<br>1.1×10^{-28}
QIN T2w	23.2 ± 9.8	29.1 ± 8.6<br>5.8×10^{-5}	28.6 ± 9.7<br>2.2×10^{-5}	51.3 ± 23.1<br>2.3×10^{-8}	57.9 ± 20.4<br>1.1×10^{-11}	-<br>-	67.5 ± 19.7<br>2.3×10^{-16}
QIN FLAIR	15.0 ± 2.4	20.8 ± 5.6<br>1.7×10^{-3}	18.3 ± 6.7<br>6.0×10^{-2}	37.3 ± 12.1<br>2.4×10^{-6}	26.3 ± 8.5<br>8.7×10^{-5}	42.6 ± 10.3<br>2.5×10^{-8}	39.9 ± 8.2<br>1.4×10^{-9}
IXI PDw	13.2 ± 4.1	13.7 ± 4.2<br>3.1×10^{-1}	18.5 ± 6.0<br>2.6×10^{-7}	34.5 ± 16.1<br>1.3×10^{-12}	52.8 ± 24.4<br>4.4×10^{-15}	43.2 ± 21.1<br>1.3×10^{-12}	42.9 ± 12.9<br>1.9×10^{-19}
FSM PDw	10.9 ± 1.7	10.3 ± 3.7<br>3.9×10^{-1}	14.8 ± 15.4<br>1.6×10^{-1}	15.6 ± 9.2<br>9.1×10^{-3}	26.9 ± 10.4<br>1.4×10^{-9}	98.3 ± 18.0<br>7.7×10^{-23}	94.4 ± 6.0<br>1.6×10^{-36}
IXI MRA	39.3 ± 13.6	42.2 ± 13.7<br>4.6×10^{-3}	53.9 ± 11.4<br>2.2×10^{-6}	39.2 ± 15.3<br>9.8×10^{-1}	36.8 ± 12.3<br>4.2×10^{-1}	-<br>-	92.4 ± 28.0<br>4.1×10^{-16}
FSM qT1	11.6 ± 3.1	61.0 ± 25.0<br>7.8×10^{-12}	132.3 ± 19.0<br>2.6×10^{-26}	89.1 ± 6.0<br>5.0×10^{-33}	84.2 ± 33.5<br>8.3×10^{-13}	91.5 ± 33.6<br>9.5×10^{-14}	130.1 ± 27.0<br>1.0×10^{-21}
ASL EPI	17.3 ± 6.5	25.3 ± 4.4<br>3.5×10^{-7}	18.9 ± 5.8<br>1.7×10^{-1}	32.7 ± 8.8<br>8.2×10^{-13}	55.3 ± 23.3<br>2.9×10^{-12}	52.9 ± 11.5<br>6.3×10^{-19}	23.8 ± 6.9<br>3.7×10^{-7}
Infant T1w	38.7 ± 22.2	44.1 ± 20.0<br>9.9×10^{-2}	64.1 ± 19.4<br>6.4×10^{-3}	78.8 ± 26.7<br>1.7×10^{-4}	68.6 ± 40.8<br>1.0×10^{-2}	65.1 ± 27.6<br>7.5×10^{-3}	60.5 ± 16.4<br>2.6×10^{-3}
IXI DWI	17.3 ± 5.2	23.0 ± 5.3<br>1.6×10^{-5}	21.5 ± 3.5<br>1.4×10^{-4}	33.5 ± 6.7<br>8.1×10^{-13}	45.1 ± 24.6<br>1.1×10^{-6}	37.9 ± 9.0<br>1.2×10^{-11}	36.4 ± 7.5<br>2.2×10^{-16}
CIM PET	12.0 ± 2.0	15.7 ± 5.2<br>4.7×10^{-3}	49.9 ± 17.2<br>2.6×10^{-8}	20.2 ± 12.5<br>1.2×10^{-2}	147.3 ± 25.3<br>7.2×10^{-14}	51.8 ± 9.6<br>5.4×10^{-12}	72.6 ± 16.0<br>8.1×10^{-13}
CIM CT	12.3 ± 2.5	24.7 ± 4.7<br>5.9×10^{-10}	123.9 ± 13.7<br>1.0×10^{-18}	58.0 ± 7.2<br>7.1×10^{-16}	155.7 ± 22.3<br>3.0×10^{-15}	105.1 ± 20.7<br>2.6×10^{-12}	132.9 ± 24.0<br>1.5×10^{-14}

Table S1. Skull-stripping accuracy across datasets, as measured by the mean Hausdorff distance (± SD) between computed and ground-truth binary brain masks. p-values comparing baseline with SythStrip results are presented below each score. Across each dataset, SynthStrip significantly outperforms most baselines except those with p-values in orange ($p > 0.05$).
