# Supplementary material for: SynthStrip: skull-stripping for any brain image
Source: Neuroimage. Author manuscript; Available in PMC 2022 Oct 15. (PMC9465771; doi:10.1016/j.neuroimage.2022.119474)
Supplement: 1 [file NIHMS1833287-supplement-1.zip › mmc1/supp_table_2.pdf]

Volume Difference (%)

	SynthStrip	ROBEX	BET	3DSS	BEaST	FSW	DMBE
IXI T1w	2.8 ± 1.9	5.8 ± 1.9<br>2.0×10^{-11}	5.2 ± 6.0<br>1.1×10^{-2}	7.0 ± 3.2<br>5.9×10^{-10}	11.9 ± 2.2<br>3.2×10^{-26}	16.0 ± 11.7<br>1.4×10^{-9}	6.9 ± 3.6<br>1.1×10^{-9}
FSM T1w	1.1 ± 1.0	7.5 ± 1.5<br>6.7×10^{-21}	104.7 ± 41.7<br>2.2×10^{-17}	5.7 ± 4.4<br>4.1×10^{-7}	14.5 ± 1.2<br>1.4×10^{-35}	12.5 ± 10.0<br>3.3×10^{-8}	7.5 ± 4.1<br>2.3×10^{-11}
ASL T1w	3.2 ± 1.6	4.1 ± 1.5<br>6.1×10^{-3}	8.6 ± 2.4<br>5.4×10^{-18}	9.0 ± 4.8<br>5.3×10^{-9}	19.4 ± 7.0<br>1.9×10^{-17}	8.5 ± 2.4<br>6.3×10^{-12}	8.3 ± 4.0<br>7.7×10^{-9}
QIN T1w	1.9 ± 1.5	8.2 ± 13.1<br>6.8×10^{-4}	6.4 ± 5.2<br>4.0×10^{-8}	8.0 ± 7.3<br>6.8×10^{-8}	17.6 ± 22.2<br>4.3×10^{-6}	83.2 ± 185.3<br>2.3×10^{-3}	15.5 ± 11.6<br>1.5×10^{-11}
IXI T2w	2.6 ± 1.3	17.0 ± 6.8<br>2.6×10^{-19}	8.7 ± 10.4<br>2.7×10^{-4}	3.8 ± 3.2<br>2.5×10^{-2}	80.4 ± 143.0<br>3.9×10^{-4}	-<br>-	96.2 ± 5.0<br>8.9×10^{-64}
FSM T2w	1.1 ± 0.9	11.9 ± 4.2<br>2.2×10^{-16}	5.7 ± 4.0<br>1.6×10^{-7}	4.0 ± 3.2<br>1.3×10^{-5}	70.2 ± 95.7<br>1.4×10^{-4}	-<br>-	96.0 ± 5.3<br>1.7×10^{-45}
QIN T2w	3.0 ± 2.9	73.3 ± 216.1<br>5.2×10^{-2}	57.7 ± 219.1<br>1.3×10^{-1}	172.9 ± 353.4<br>5.2×10^{-3}	91.5 ± 213.6<br>1.5×10^{-2}	-<br>-	31.2 ± 22.4<br>1.7×10^{-9}
QIN FLAIR	1.8 ± 1.0	9.6 ± 3.8<br>1.7×10^{-6}	2.3 ± 2.3<br>4.0×10^{-1}	4.8 ± 2.0<br>1.7×10^{-5}	17.0 ± 7.3<br>3.5×10^{-7}	34.6 ± 19.6<br>5.9×10^{-6}	13.0 ± 4.3<br>2.3×10^{-8}
IXI PDw	2.3 ± 1.1	7.2 ± 3.3<br>9.2×10^{-14}	4.4 ± 3.1<br>1.2×10^{-5}	3.6 ± 3.0<br>1.1×10^{-2}	52.0 ± 83.3<br>1.2×10^{-4}	105.0 ± 229.4<br>2.9×10^{-3}	8.9 ± 5.7<br>1.2×10^{-10}
FSM PDw	3.0 ± 1.8	4.7 ± 3.3<br>4.4×10^{-2}	7.5 ± 22.7<br>2.8×10^{-1}	4.8 ± 2.4<br>4.0×10^{-5}	15.5 ± 13.6<br>1.2×10^{-5}	102.3 ± 45.2<br>3.6×10^{-13}	37.4 ± 14.1<br>1.9×10^{-14}
IXI MRA	1.7 ± 1.1	44.4 ± 12.1<br>7.2×10^{-29}	52.7 ± 16.6<br>1.5×10^{-26}	8.6 ± 2.1<br>3.6×10^{-29}	111.6 ± 296.2<br>1.2×10^{-2}	-<br>-	88.4 ± 15.1<br>5.3×10^{-39}
FSM qT1	1.1 ± 1.0	173.8 ± 191.0<br>2.0×10^{-5}	243.2 ± 116.4<br>8.5×10^{-13}	262.2 ± 139.2<br>1.1×10^{-11}	98.8 ± 166.6<br>2.7×10^{-3}	153.8 ± 193.5<br>1.2×10^{-4}	76.6 ± 15.2<br>2.0×10^{-23}
ASL EPI	5.8 ± 2.3	24.4 ± 14.1<br>4.6×10^{-10}	7.2 ± 4.3<br>1.5×10^{-1}	3.9 ± 2.8<br>8.3×10^{-2}	77.6 ± 93.1<br>1.4×10^{-5}	81.8 ± 27.4<br>1.6×10^{-20}	6.5 ± 3.4<br>1.8×10^{-1}
Infant T1w	7.4 ± 3.5	34.7 ± 65.7<br>1.2×10^{-1}	142.4 ± 154.6<br>3.7×10^{-3}	106.2 ± 124.6<br>7.0×10^{-3}	228.0 ± 358.7<br>3.0×10^{-2}	259.4 ± 294.2<br>4.6×10^{-3}	19.8 ± 11.8<br>3.7×10^{-4}
IXI DWI	5.5 ± 3.5	32.0 ± 18.0<br>1.2×10^{-8}	10.8 ± 4.5<br>5.4×10^{-12}	17.0 ± 3.9<br>1.0×10^{-18}	37.8 ± 33.0<br>5.6×10^{-6}	84.2 ± 167.9<br>1.3×10^{-2}	31.9 ± 4.2<br>2.1×10^{-31}
CIM PET	3.6 ± 3.6	23.3 ± 14.1<br>2.0×10^{-6}	58.1 ± 29.1<br>7.4×10^{-8}	8.3 ± 20.1<br>2.7×10^{-1}	174.9 ± 275.5<br>1.4×10^{-2}	120.3 ± 207.9<br>2.4×10^{-2}	29.2 ± 20.9<br>5.4×10^{-5}
CIM CT	11.5 ± 2.0	70.5 ± 7.7<br>6.3×10^{-20}	243.9 ± 40.4<br>4.7×10^{-16}	132.0 ± 16.2<br>2.6×10^{-18}	157.4 ± 288.4<br>4.0×10^{-2}	313.6 ± 292.4<br>2.4×10^{-4}	64.3 ± 24.5<br>1.2×10^{-8}

Table S2. Skull-stripping accuracy across datasets, as measured by the mean percent difference ($\pm$ SD) in volume between computed and ground-truth binary brain masks. p-values comparing baseline with SythStrip results are presented below each score. Across each dataset, SynthStrip significantly outperforms most baselines except those with p-values in orange, for which $p > 0.05$.
